# Report of Proceedings of the Illinois State Board of Health, Quarterly Meeting, Nov. 20–21, 1884

**Published:** 1885-01

**Authors:** 


REPORT OF PROCEEDINGS OF THE ILLINOIS STATE BOARD OF HEALTH. QUARTERLY MEETING, NOVEMBER 20–21, 1884.

The regular quarterly meeting of the State Board of Health of the State of Illinois was held in the rooms of the board in the capitol building at Springfield, November 20 and 21, 1884.

Present, the Hon. Newton Bateman, President of the board, and Drs. Clarke, MacKenzie, Kreider and Rauch.

After the reading and approval of the minutes of the last meeting, the board went into executive session, for the consideration of charges against certain practitioners under the Medical Practice Act.

At the evening session, the Secretary presented the following:

*Quarterly Report of the Secretary.*—During the quarter ending September 30, 1884, there were received in the Secretary's office 1,623 communications, letters, reports, etc., and 3,472 letters, postals and other written communications were sent out. Of printed matter there were distributed 2,689 copies of the Fifth Annual Report, and upwards of 200,000 copies of other printed matter—the mail and express packages sent out during the quarter aggregating 8,982 pounds' weight, or over four tons.

Among the more important written and printed documents distributed were those concerning the vaccination of school children, sent to about 12,000 school districts, through the County Superintendents:

Concerning the Sanitary Inspection of Public Buildings—especially of Almsmouses, Jails and similar Institutions—sent to County Commissioners, Boards of Supervisors and other officers:

Concerning the Sanitation of Railway Buildings, Grounds and Travel, sent to the general officers of thirty-three railway companies operating in this State:

Concerning Preventable Diseases, sent to localities in which Small-Pox, Scarlet Fever, Diphtheria or Typhoid Fever appeared.

In connection with these latter circulars, the blanks for Reports of Epidemic Diseases have been revised, and a new edition has been partly printed, together with a circular of instructions for their use.

A pamphlet of 51 octavo pages has also been prepared printed and distributed, containing the Public Health Laws of Illinois; the Form of an Ordinance for the Protection of the Public Health, suggested for adoption by communities which have no health organization, and for substitution for existing health ordinances which have been found defective or inoperative; Rules and Regulations concerning Contagious Diseases; concerning vaccination; concerning the Sanitation of smaller Cities and Towns; and concerning the Principles and Practice of General Sanitation.

*Medical Practice.*—State certificates, entitling to practice medicine and surgery in Illinois, were issued to 105 graduates, 88 of which were granted unconditionally upon the diplomas of medical colleges in good standing; 6, upon examination in omitted branches, to graduates of colleges which had not fully complied with the schedule of minimum requirements of the board; and 11, upon presentation of evidence of proper preliminary education, to graduates of colleges otherwise in good standing, but which had not yet enacted a matriculation examination at the beginning of the session of 1883-84. There were also issued 8 duplicate certificates upon affidavits of the loss or destruction of the originals, and one certificate based upon length of practice in the State.

To midwives, 6 certificates have been issued upon the diplomas or licenses of recognized schools of midwifery, and 3 upon satisfactory examination.

*Quacks and Disreputables* —With the exception of those in Chicago, the fraudulent advertising quacks and disreputable specialists seem to have been pretty well weeded out of the State. For the first time since the passage of the Medical-Practice Act there have been no complaints received concerning this class, except as above indicated.

After repeated attempts, a grand jury was found which indicted eleven of the more prominent of those in Chicago during the month of July; but thus far none of the number has been convicted.

The noted quack, R. C. Flower, of Boston, Mass., has finally abandoned his efforts to secure a foothold in Chicago. By means of insidious and plausibly-worded advertisements, frequently of over a column in length, he succeeded in doing quite a thriving business for a time, and charged the most exorbitant fees. Unable to comply with the law and obtain a State certificate, and being refused an itinerant license, he was compelled to make appointments with his patients at Michigan City, in Indiana, and at Davenport, in Iowa, only venturing to stay in the State for a day or two at a time, and leaving before his arrest could be effected. Some of his dupes and victims have lodged complaint against him, and are now anxious to secure his arrest and punishment.

The suit of Frank B. Smith, one of the "K. and K. Surgeons," against the Secretary of the Board, for $50,000 damages, alleged to have been sustained by the revocation of his certificate by the board on charges of unprofessional and dishonorable conduct, and which suit was brought at Detroit, Mich., in the United States District Court, has been dismissed and the plaintiff mulct in costs.

*The Public Health.*—Small-pox, noted as exisiting in isolated localities in the southern portion of the State at the date of the last report, was practically extinct at the close of the quarter, with the exception of a few cases in Marshall county, the contagion of which was introduced from Indiana. Reports of a serious epidemic of the disease in Ballard county, Ky., threatening Cairo and the line of the Illinois Central Railroad, led me to visit the locality early in August, after communicating with the Secretary of the Kentucky State Board of Health.

The precautions necessary to protect the threatened region of our own State were instituted, and these were efficiently supplemented by the action of the management of the Illinois Central, under the direction of the Superintending Surgeon, Dr. John E. Owens.

Notwithstanding the freedom of the State from this disease at the present time, and its subsidence abroad, the necessity for vaccination and re-vaccination in all localities where there are still unprotected individuals is likely to become apparent upon the approach of cold weather; and it is incumbent upon local health authorities to secure the fullest protection in season.

There has been a diminution in the prevalence of scarlet fever during the quarter; but toward its close there is noted an increase of diphtheria and of typhoid fever. The demand for the preventable-disease circulars of the board has, in consequence, been much greater than usual, and that on diphtheria has been re-printed, in part or whole, by many newspapers.

In response to a telegram from Dr. Salmon, the veterinary expert of the Department of Agriculture at Washington, I went to Peoria on the night of the 17th of August, and on the following day examined some cattle suspected of being infected with pleuro-pneumonia. The post-mortem examination of one of these animals confirmed the diagnosis, and since that date the disease has been detected in several other localities. Occasional cases continue to be reported, but the State veterinarian believes the outbreak is in a fair way to be suppressed. The necessity for additional legislation on the subject of the contagious diseases of animals, already suggested from time to time in these reports, is emphasized by this outbreak.

*Sanitary Inspection and Work.*—The results of the efforts made in accordance with the instructions of the board at the last quarterly meeting, to secure a general inspection and improvement of sanitary conditions have been very gratifying. Reports from 230 cities, towns and villages have thus far been received in reply to the circulars sent out, and an immense amount of work has already been accomplished in remedying the defects disclosed by the inspection. In many localities it is known that reports are deferred until the completion of work already being pushed forward in anticipation of the advent of cholera next year.

I have personally inspected a number of the State institutions, and find them in as good sanitary condition as could be expected in view of obvious faulty construction, or location, from a hygienic standpoint. Such suggestions of improvement as I have found it necessary to make have been carried out as far as practicable.

Responses to the special circular concerning railway buildings, grounds and travel, have been received from sixteen companies, comprising the more important of all the roads operating in Illinois.

On the whole, there is cause for congratulation in the progress already made in this effort of the board to secure the best attainable sanitary condition of the State as the most efficient and valuable mode of warding off an epidemic of Asiatic cholera.

It is to be wished, however, that the newspaper press, especially in the smaller cities and towns, would devote some of their space to articles urging the fundamental importance of individual sanitary effort. Without this, boards of health and health officers are to a great extent inadequate to cope with some of the more serious evils. A large portion of the community needs to be taught that personal cleanliness and cleanliness of the household and premises are among the highest results of sanitary science, and that, of themselves, they constitute the best safeguards against contagion and preventable disease.

*The Cholera.*—Soon after the adjournment of the last meeting of the board, the spread of Asiatic cholera in Europe and the indications of its possible cis-Atlantic extension became so threatening that on the 17th of July I addressed a communication to the Hon. Erastus Brooks, of New York, chairman of the National Conference of State Boards of Health, suggesting that a session of the conference be held in Washington City, with the view of securing concert of action on the part of all those charged with the administration of public health affairs, of devising some general and efficient system of supervision and notification at all seaports, and of ascertaining authoritatively the plans of the general government with reference to measures for the prevention and limitation of the threatened epidemic. To this meeting it was proposed to invite the health officers and quarantine authorities of all seaports and boundary towns, the health authorities of important inland cities—especially those in States having no State Boards of Health—and the health authorities of the Dominion of Canada.

The suggestion was favorably received, and the time of the meeting was fixed for the 7th of August; but, before that date the President and the members of the cabinet with whom it was desired to confer, had left Washington, so that the chief reason for deciding upon the national capital as the place of meeting was destroyed, and this fact, coupled with more favorable news from Europe, led me to propose a postponement to the regular period of meeting, namely, during the session of the American Public Health Association. The

National Conference accordingly met, in the city of St. Louis, on the 13th of October, delegates from State Boards of Health and from various health organizations in twenty States, and representatives of the Provincial Board of Health of Ontario, and of the government of the Dominion of Canada, being in attendance. The session, which was continued on the 14th and 15th, was devoted entirely to the consideration of the questions above indicated, and the report, formulated in the discussions, addresses and papers, and adopted by the conference, was subsequently endorsed by the American Public Health Association, ordered to be printed, and copies forwarded to the President of the United States and his cabinet, to each of the Senators and Representatives in the national Congress, to the health officers of cities, to the various State Boards of Health, and to the officers of the Dominion of Canada and of the Provincial Board of Health of Ontario.

Copies of the report of the proceedings of the conference, including the text of a paper by Dr. C. W. Chancellor, Secretary State Board of Health of Maryland, "Can Epidemic Diseases be excluded by Sanitary Cordons?" of a memorandum of "Quarantine and Sanitary Methods formulated by the National Board of Health in re-Asiatic Cholera," prepared by Dr. Charles Smart, U. S. A., member of the N. B. H.; and of my address at the opening of the conference—"Practical Recommendations for the Exclusion and Prevention of Asiatic Cholera in North America," have already been furnished to the members of the board; so that it is not necessary at this time to do more than refer to the illustration, furnished by recent developments, of one of the points made in my address, to-wit: "That we may not know how widely spread the disease now is on the European continent, and we do not know how soon its arrival on our own shores may be announced." The proof of systematic and persistent suppression of damaging information by European authorities, which I then submitted, and since corroborated by the disclosure of the existence of cholera in Paris for months before the fact was reported, justifies us in suspecting a much wider extension of the area of infection than is acknowledged or known to exist.

The action of the board has already anticipated all the practical measures which have been recommended in the interim since our last meeting, and I do not know that there remains anything more for the board to do in its official capacity beyond a formal endorsement of the report of the national conference.

*Recommendations and Suggestions.*—The following recommendations and suggestions are respectfully submitted:

*First*—That a thorough sanitary survey of the State be inaugurated not later than the 1st of January, 1885, under the direction of the board.

*Second*—That a committee of the board be appointed to prepare revisions and amendments of the laws of the State regulating the practice of medicine, and concerning the protection of the public health. The defects of the statutes concerning both these subjects are patent, and should be remedied as speedily as practicable.

*Third*—That action be taken in anticipation of the forthcoming meeting of the National Conference of the State Board of Health, with reference to the subject of Asiatic cholera.

JOHN H. RAUCH, *Secretary*.

Upon the conclusion of the reading, on motion of Dr. Clark the report was accepted, the recommendations and suggestions were taken up for consideration, and the following action was had:

*Sanitary Survey of the State.*—Dr. Kreider submitted the following resolution, which was adopted:

*Resolved*, That the Secretary be authorized to prepare the necessary blanks and instructions, and to distribute the same to the proper authorities of counties, townships and municipalities, for a thorough and systematic sanitary survey of the State, to be begun by January 1, 1885, or as soon thereafter as practicable.

The Secretary explained that it was proposed to begin work in the southern portions of the State and to work northward as rapidly as the weather would permit, so that by the 1st of May the sanitary condition of every dwelling, in all of its parts, of all premises, outhouses, wells, cisterns and other belongings, should be made known, the remedy of defects be pushed, and the authority of the State Board be exerted wherever necessary to supplement the efforts of local authorities in the preparation of the State to resist the threatened invasion of Asiatic cholera.

*National Conference on Asiatic Cholera.*—With reference to the forthcoming meeting of the National Conference of State Boards of Health, to be held in the city of Washington, December 10, prox., to consider the subject of Asiatic cholera, Dr. Clark offered the following preamble and resolutions, which were adopted:

WHEREAS, The members of this board, having carefully considered the able and exhaustive paper upon the Exclusion and Prevention of Asiatic Cholera in North America, prepared by the Secretary of the Board, find the argument set forth abundantly supported by incontestable facts duly cited in the text, and believe its conclusions and recommendations to be comprehensive, practical and sufficient; and,

WHEREAS, This subject is the most important of any which now demands the attention of those charged with the protection of the public health—involving, as it does, the prevention of a great sacrifice of human life, of an immense money expenditure, and of serious and widespread injury to commerce, manufactures and all other industries—therefore, be it

*Resolved,* That a committee be appointed to draft a formal expression of the views of the State Board of Health of the State of Illinois concerning the measures which should be adopted and enforced by municipalities, State and the National Government for the protection of the country against an invasion of Asiatic cholera.

*Resolved,* That the action of the National Conference of the State Boards of Health, had at St. Louis, October 13–15, 1884—on the subject of Asiatic cholera—be, and the same hereby is, approved and endorsed by this Board.

*Resolved,* That the Secretary of this Board be authorized to attend the forthcoming meeting of the National Conference in Washington, D. C., and to present to said Conference the action of this Board as above indicated.

On motion of Dr. Mackenzie, the chair was authorized to appoint the committee, to consist of five members, including the President as chairman of the committee. Drs. Haskell, Clark, Mackenzie and Rauch, and the Hon. Newton Bateman were thereupon appointed as members of the committee.

*Revision of the Laws.*—On motion of Dr. Kreider, the President appointed a committee, consisting of Drs. Rauch, Haskell, Kreider, Clark and Mackenzie, to prepare revisions and amendments of the laws regulating the practice of medicine and concerning public health, to be submitted to the next General Assembly.

During the executive session of the Board the case of Dr. Ed. S. McLeod, of Chicago, was considered. The following extract from the formal notification citing McLeod to appear before the Board, show cause why his certificate should not be revoked for "unprofessional and dishonorable conduct," sufficiently explains this case.

"The charges against you are, that you ply your vocation by means of fraudulent and deceptive advertisements under assumed names, to wit: under the alias of 'Dr. James' and

'Dr. Lucas.' That in order to secure patients, you hold out inducements and promises, and make suggestions, which, in themselves tend to promote crimes and immorality. That you publish and distribute, through the U. S. mails and otherwise, to all classes of the community, including the youth of both sexes, obscene circulars and pamphlets, for which you have already been once indicted in the U. S. District Court at Chicago, when you pleaded guilty, were fined $500 and costs, and your plates and circulars were seized and destroyed by the United States authorities. That such fraudulent, deceptive and demoralizing practices constitute 'unprofessional and dishonorable conduct' within the meaning and intent of the statute, which was enacted for the protection of the people from the ignorant and unscrupulous under the guise of medical practice."

Upon mature deliberation and careful consideration of the evidence offered in support of the charges, the certificate of Dr. Ed. S. McLeod was ordered to be revoked.

After the transaction of the regular routine business and auditing of accounts and bills, amounting to $2,876.55, the Board adjourned.

